# The Risk of Acute Kidney Injury in Hepatitis B Virus-Related Acute on Chronic Liver Failure with Tenofovir Treatment

**DOI:** 10.1155/2020/5728359

**Published:** 2020-05-18

**Authors:** Kai Zhang, Su Lin, Mingfang Wang, Jiaofeng Huang, Yueyong Zhu

**Affiliations:** Liver Research Center, The First Affiliated Hospital of Fujian Medical University, Fuzhou, China

## Abstract

**Aims:**

Tenofovir (TDF) is an antiviral drug with potential risk of kidney injury. The study is aimed at comparing the incidence of acute kidney injury (AKI) between TDF and entecavir (ETV) treatment in hepatitis B virus- (HBV-) related acute on chronic liver failure (ACLF).

**Methods:**

Treatment-naive patients with HBV-related ACLF were included. Propensity score matching was used to balance the baseline characteristics between ETV and TDF groups. The risk of AKI and the efficacy of TDF and ETV were compared.

**Results:**

A total of 95 cases with HBV-related ACLF were included in this study, with 74.74% of male and a mean age of 47.01 ± 14.71 years. The antiviral therapy was initiated within 2 days after admission, with 39 cases on the TDF group and 56 on the ETV group. Patients in the TDF group had higher AST, hemoglobin, and serum sodium levels and lower MELD-Na score. After propensity matching, 39 cases of TDF and 39 of ETV were included in the final analysis. No difference was found in the changes of creatinine and cystatin C from baseline to 4 weeks after treatment between ETV and TDF groups. AKI was developed in 1 (2.56%) patient in the ETV group and 2 (5.13%) in the TDF group within one month (*P* = 0.556). Survival analysis revealed no significant difference in the 6-month mortality between the two groups (*P* = 0.813). Cox analysis showed that the type of antiviral drug or the development of AKI was not an independent risk factor for the outcomes.

**Conclusions:**

Compared to ETV, TDF did not increase the risk of AKI nor the mortality in patients with HBV-related ACLF in the short time.

## 1. Introduction

Hepatitis B virus (HBV) is a major health problem with 3.5% of the population being chronically infected globally [1]. Patients with chronic HBV infection may suffer from various hepatic complications, such as cirrhosis, liver failure, and hepatocellular carcinoma [2]. Acute on chronic liver failure (ACLF) is defined as a precipitating event in a patient with chronic liver disease, leading to jaundice and coagulopathy complicated by clinical ascites and/or encephalopathy [3]. Patients with ACLF due to HBV reactivation (HBV-ACLF) have extremely poor prognosis, with a reported short-term mortality ranging from 29.7% to 40% within 28 days [4–6]. Acute kidney injury (AKI) is common in ACLF and may develop within a very short period and lead to a poor outcome in ACLF [7].

The management of HBV-ACLF includes antiviral therapy, artificial liver support system, alternative therapies, and liver transplantation [8]. The antiviral therapy is the most evident treatment among them. Currently, tenofovir (TDF) and entecavir (ETV) are both recommended as the first-line antiviral agents for their potent antiviral activity and high genetic barrier for drug resistance [9, 10]. However, TDF has also been demonstrated to have potential kidney toxicity by several observational studies and case reports [11–14]. It is unclear whether or not the use of TDF may increase the risk of AKI in ACLF. The aim of this study was to compare the risk of AKI and the mortality between ETV and TDF groups in HBV-ACLF.

## 2. Patients and Methods

### 2.1. Patients

We retrospectively reviewed cases of HBV-related ACLF hospitalized in the First Affiliated Hospital of Fujian Medical University between January 2016 and November 2018. Treatment-naive patients who were diagnosed with ACLF and received TDF or ETV therapy after hospitalization were included in this study. The exclusion criteria were as follows: (1) patients with kidney injury on baseline; (2) patients with nucleotide treatment other than ETV or TDF; (3) patients with malignant tumor; (4) patients concomitant with other liver diseases such as alcoholic liver disease, autoimmune hepatitis, drug-induced liver injury, or other viral infections (hepatitis A, C, and E virus or HIV infection); (5) patients with missing data; and (6) patients who died or were lost to follow-up within one week after admission.

The diagnosis of ACLF was based on the definition by the Asian Pacific Association for the Study of the Liver (APASL) [3]: jaundice (a serum bilirubin level of ≥5 mg/dL) and coagulopathy (an international normalized ratio (INR) of ≥1.5 or prothrombin activity of <40%). The definition of AKI was based on the criteria by the International Club of Ascites (ICA), which is an increase in serum creatinine (sCr) ≥ 0.3 mg/dL (≥26.5 *μ*mol/L) within 48 hours or a percentage increase in sCr ≥ 50% from baseline which is known, or presumed, to have occurred within the prior 7 days. A value of sCr obtained in the previous 3 months, when available, can be used as baseline sCr. In patients with more than one value within the previous 3 months, the value closest to the admission time to the hospitalization was used [15].

### 2.2. Treatments

During hospitalization, all patients received supportive treatments including nutrition support, albumin, and other medications that aimed to protect the liver. In patients with liver failure, plasma exchange was given if necessary. Antiviral therapy with TDF or ETV was started immediately when HBV-DNA was detected.

### 2.3. Data Collection and Follow-Up

The clinical and laboratory data were collected on admission, including the presence of ascites or hepatic encephalopathy (HE), the presence of underlying cirrhosis, total bilirubin (TBIL), albumin, alanine aminotransferase (ALT), aspartate transaminase (AST), international normalized ratio (INR), serum creatinine (sCr), cystatin C, glomerular filtration rate (GFR), serum sodium (Na), hemoglobin, platelets, white blood cell (WBC), Child-Turcotte-Pugh (CTP) score, model for end-stage liver disease (MELD) score, chronic liver failure-sequential organ failure assessment (CLIF-SOFA), hepatitis B surface antigen (HBsAg) levels, hepatitis B e antigen (HBeAg), and HBV DNA levels. Patients were divided into ETV and TDF groups according to the antiviral treatment.

The renal function was reexamined in all survival patients on 4 weeks after antiviral treatment. The survival status was followed up until 2019. For patients being transferred to local hospital, the survival status was collected upon phone contact. The primary outcome was the incidence of AKI within 1 month; the secondary outcome was death or liver transplantation.

### 2.4. Statistical Analyses

The continuous variables were reported as mean ± standard deviation or medium (interquartile rage), while categorical variables were reported as percentage. The Student *t*-test was used for the comparisons of continuous variables, and the chi-squared test was used for the comparison of categorical variables [16]. Propensity score matching (PSM) analysis was performed to minimize the probability of selection bias [17]. The Cox proportional hazard model was used to analyze the risk factors of mortality. The log-rank test was used to compare the risks between groups. All statistical analyses were performed using SPSS software version 24.0 (SPSS Inc., Chicago, USA).

## 3. Results

### 3.1. Patient Characteristics

**Figure 1:**
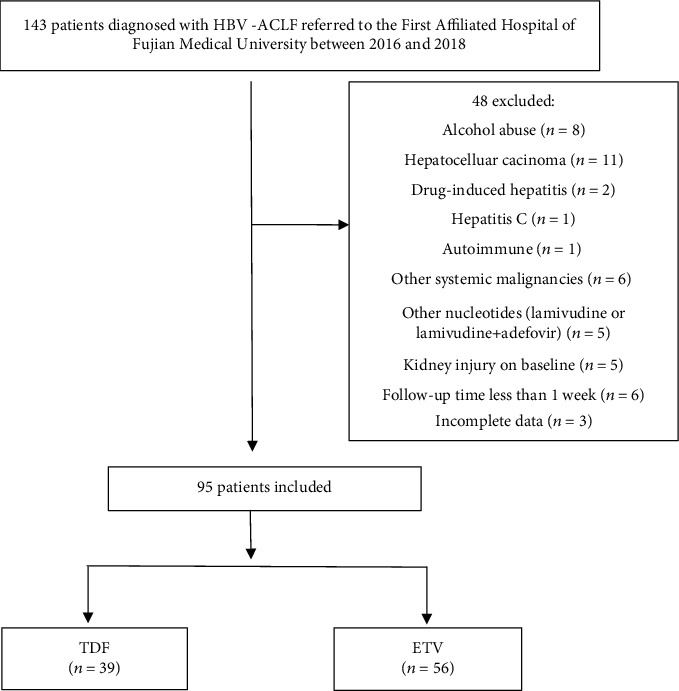
Flow chart of patient selection.

A total of 143 patients were diagnosed with ACLF during the study period, among whom 48 patients were excluded due to various reasons (Figure 1). Ninety-five cases were eligible for the final analysis, including 56 cases with ETV therapy and 39 cases with TDF therapy (Figure 1). The average age was 47.01 ± 14.71 years old, and 71 (74.74%) of them were male. The median follow-up time of the overall population was 531 days (range 14-1207 days). There were 20 patients who died during this time period, with a median survival time of 26 days. The baseline characteristics are shown in Table 1. Patients in the TDF group had higher AST, hemoglobin, and serum sodium levels and lower MELD-Na score. There was no difference in other baseline characteristics, including age, sex, HBV DNA levels, MELD score, and the presence of underlying cirrhosis.

We performed PSM to balance the baseline factors. After PSM, there were 39 cases with ETV treatment and 39 cases with TDF treatment that were finally included. The baseline characteristics were comparable between the two groups after PSM. There were 15 patients in this PSM cohort who died during this follow-up, with a median survival time of 35 days.

### 3.2. Virological and Serological Responses in TDF and ETV Groups

Significant reductions in HBV-DNA, bilirubin, and ALT were observed in both TDF and ETV groups after two weeks of treatment, with no difference in the reduction level between the two groups (Table 2). The HBV-DNA undetectable rate after 2 weeks of antiviral therapy was 28.21% (11/39) in the ETV group and 35.90% (14/39) in the TDF group (*P* = 0.467).

### 3.3. The Dynamic Changes of Renal Function in TDF and ETV Groups

Slight increases in sCr were found in both TDF and ETV groups after treatment. However, no significant difference in the change of sCr within 2 weeks or 4 weeks was found within each group or between two groups. Significant difference in the change of cystatin C within 2 weeks or 4 weeks was found within each group, but no significant difference in the dynamic changes of cystatin C between ETV and TDF groups (Table 3). Patients were followed up for 1 month, and AKI was developed in 1 (2.56%) patient in the ETV group and 2 (5.13%) patients in the TDF group. This difference was not statistically significant (*P* = 0.556). All of these 3 patients with AKI had cirrhotic background and pneumonia on admission. Two of them had diabetes. The patients with AKI in the ETV group died at 8 weeks after admission. The other two patients in the TDF group survived (Table 4).

### 3.4. The Mortality in Overall Study Population and Predictors for Mortality

A total of 15/78 (19.23%) patients died within 6 months. Survival analysis revealed no significant difference in the 6-month mortality between two groups (*P* = 0.813). The results of univariate analysis showed that age, HE, HBeAg positive, MELD score-Na, CTP score, and SOFA score were related to the overall mortality.

Before multivariate analysis, collinearity diagnostics was conducted to assess the sources of collinearity among MELD-Na, CTP, and SOFA scores. The result showed that the tolerance of all variables > 0.1 and the variance inflation factor < 5, indicating limited collinearity among the above variables. As the presence of cirrhosis, HBV DNA, and AKI and gender had been reported to be important predictive factors for the prognosis of ACLF [18–21], those were included in multivariate analysis as well.

**Figure 2:**
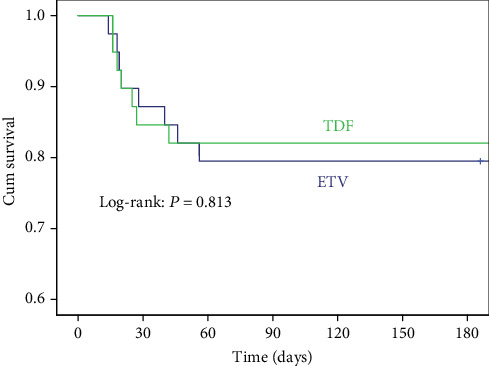
Cumulative survival of ETV and ETV within 6 months.

The results of multivariate Cox regression analysis showed that the age (HR = 1.103, 95% CI: 1.038-1.172, *P* = 0.002), CTP score (HR = 1.990, 95% CI: 1.210-3.271, *P* = 0.007), SOFA score (HR = 3.000, 95% CI: 1.366-3.171, *P* < 0.001), and cirrhosis (HR = 47.232, 95% CI: 5.538-402.802, *P* < 0.001) were independent risk factors for mortality (Table 5). The types of antiviral drug and the development of AKI were not independently associated with the outcome (Figure 2 and Table 5).

## 4. Discussion

This study compared the impact of TDF and ETV in renal function in patients with HBV-ACLF. The results showed that TDF did not increase the risk of AKI nor the mortality in patients with HBV-related ACLF within 6 months.

Both TDF and ETV are currently recommended as the first-line treatment for chronic hepatitis B (CHB) for their high efficacy and low resistance rate [9, 22–24]. Previous studies have demonstrated that TDF and ETV have similar effectiveness in treatment-naive CHB patient [25–27]. However, some reports indicate that TDF might lead to a higher incidence of AKI compared to ETV in CHB patients [28, 29]. As AKI is common in ACLF [30], renal injury associated with TDF use has raised some concerns [31]. However, in this single-center study, we found that the use of TDF did not increase the risk of AKI within one month of treatment. This might be due to the short follow-up period of this study. As reported previously, renal injury associated with TDF use usually develops after at least one year of treatment. A recent real-world study from Korea showed that TDF therapy did decrease overall renal function in CHB patients during the first two years of TDF use [13]. Therefore, long-term follow-up might be helpful to access the renal impairment in ACLF patients with different antiviral therapies.

It is worth noticing that all three patients suffering from AKI had bacterial infection and two of them had comorbidities like diabetes and hypertension. Hypertension and diabetes are both well-known risk factors for chronic kidney injury. The bacterial infection is also a main trigger for AKI in liver failure [32]; thus for patients who had AKI in this cohort, the impact of the other complication/comorbidities might overwhelm the influence of antiviral drugs. Prospective studies with longer follow-up period are greatly needed to reveal the real relationship between AKI and TDF in ACLF patients.

Cystatin C is a sensitive marker for renal impairment [33]. In this study, no significant difference in the change of sCr within 1 month was found in both TDF and ETV groups, while there was significant difference in the change of cystatin C in both groups. Cystatin C levels may be more sensitive for evaluating the renal impairment in ACLF [34]. However, in terms of the impact of different antiviral drugs on renal function, the changes of cystatin C were similar as those of sCr, which further consolidated that TDF had limited influence on renal function in an ACLF population in a short-term period.

The efficacy of different antiviral drugs in ACLF remains controversial. Wan et al. [35] showed that TDF was superior to ETV in the treatment of HBV-ACLF; however, more studies showed no difference between these two groups [27, 36, 37]. The results of our study were in consistence with most studies showing that TDF was not superior to ETV regarding the HBV DNA suppression or mortality.

There are several limitations of this study. Firstly, the data of HBV-DNA levels, liver function, and kidney function is largely missing after 3 months because most survival patients were transferred to a local hospital after recovery; thus, the long-term changes of renal function were unclear. Secondly, the incidence rate of AKI was low and the sample size relatively small, which may easily lead to false-negative results. Further study with larger sample size is needed to guarantee the results.

In summary, our study showed that compared with ETV, TDF did not increase the risk of AKI nor the mortality in patients with HBV-related ACLF within a short-term period.

## Figures and Tables

**Table 1:** Baseline characteristics of study population.

Variable	Unmatched			Matched		
	ETV group (*n* = 56)	TDF group (*n* = 39)	*P* value	ETV group (*n* = 39)	TDF group (*n* = 39)	*P* value
Age (years)	47.80 ± 14.16	44.33 ± 15.87	0.266	45.97 ± 14.10	44.33 ± 15.87	0.631
Male, *n* (%)	42 (75.00%)	29 (74.36%)	0.944	30 (76.92%)	29 (74.36%)	0.792
Ascites, *n* (%)	44 (78.57%)	31 (79.49%)	0.914	29 (74.36%)	31 (79.49%)	0.591
HE, *n* (%)	9 (16.07%)	5 (12.82%)	0.884	7 (17.95%)	5 (12.82%)	0.530
Cirrhosis, *n* (%)	39 (69.62%)	29 (74.36%)	0.787	26 (66.67%)	29 (74.36%)	0.456
TBIL (mmol/L)	282.15 ± 131.00	259.64 ± 120.26	0.396	274.60 ± 138.61	259.64 ± 120.26	0.612
ALT (U/L)	624.61 ± 571.32	861.64 ± 691.44	0.071	724.79 ± 601.63	861.64 ± 691.44	0.354
AST (U/L)	419.04 ± 372.70	645.00 ± 629.04	0.031	490.10 ± 405.92	645.00 ± 629.04	0.200
Albumin (g/L)	30.05 (27.85-32.80)	30.00 (27.90-34.00)	0.934	29.80 (27.40-33.30)	30.00 (27.90-34.00)	0.768
INR	2.14 ± 0.89	1.96 ± 0.55	0.249	1.94 ± 0.59	1.96 ± 0.55	0.864
BUN (mmol/L)	4.33 ± 2.00	3.58 ± 1.52	0.052	4.07 ± 1.84	3.58 ± 1.52	0.203
sCr (*μ*mol/L)	59.81 ± 12.35	57.86 ± 13.87	0.474	59.23 ± 11.24	57.86 ± 13.87	0.633
Cystatin C (mg/L)	1.11 ± 0.41	1.00 ± 0.21	0.128	1.06 ± 0.26	1.00 ± 0.21	0.301
GFR (mL/min)	93.00 ± 18.71	96.72 ± 23.24	0.392	94.49 ± 19.79	96.72 ± 23.24	0.650
HBsAglog10 (ng/mL)	3.10 ± 1.08	2.98 ± 1.07	0.595	3.28 ± 1.12	2.98 ± 1.07	0.243
HBeAg-positive, *n* (%)	26 (46.43%)	22 (52.79%)	0.454	19 (48.72%)	22 (52.79%)	0.496
HBVDNAlog10 (IU/mL)	5.11 ± 2.00	5.34 ± 1.68	0.560	5.39 ± 1.95	5.34 ± 1.68	0.903
Na (mmol/L)	136.16 ± 3.70	138.12 ± 2.93	0.007	136.62 ± 3.91	138.12 ± 2.93	0.058
WBC (×10^9^/L)	6.38 ± 3.24	7.21 ± 3.57	0.247	6.68 ± 3.45	7.21 ± 3.57	0.510
HGB (g/L)	119.07 (102.25-136.50)	132.67 (119.00-147.00)	0.011	124.00 (111.00-143.00)	132.67 (119.00-147.00)	0.147
Platelets (×10^9^/L)	106.95 ± 52.22	118.97 ± 60.22	0.303	116.03 ± 53.48	118.97 ± 60.22	0.820
CTP score	10.48 ± 1.87	10.36 ± 2.12	0.766	10.18 ± 1.90	10.36 ± 2.12	0.694
MELD score	20.25 ± 6.80	18.33 ± 5.20	0.139	18.22 ± 4.94	18.33 ± 5.20	0.928
MELD-Na score	21.68 ± 7.81	18.74 ± 5.70	0.047	19.46 ± 6.15	18.74 ± 5.70	0.593
CLIF-SOFA score	7.25 ± 1.73	7.05 ± 1.96	0.603	6.97 ± 1.67	7.05 ± 1.96	0.852
Diabetes, *n* (%)	8 (14.29%)	3 (7.70%)	0.508	5 (12.82%)	3 (7.70%)	0.709
Hypertension, *n* (%)	7 (12.50%)	2 (5.13%)	0.395	4 (10.26%)	2 (5.13%)	0.671

HE: hepatic encephalopathy; TBIL: total bilirubin; ALT: alanine aminotransferase; AST: aspartate transaminase; INR: international normalized ratio; BUN: blood urea nitrogen; sCr: serum creatinine; GFR: glomerular filtration rate; HBV: hepatitis B virus; HBsAg: hepatitis B surface antigen; HBeAg: hepatitis B e antigen; WBC: white blood cell; HGB: hemoglobin; CTP: Child-Turcotte-Pugh; MELD: model for end-stage liver disease; CLIF-SOFA: chronic liver failure-sequential organ failure assessment.

**Table 2:** Index changes between ETV and TDF groups after 2-week treatment.

	ETV (*n* = 39)	TDF (*n* = 39)	*P* (ETV vs. TDF)
HBVDNA			
Before treatment	5.39 ± 1.95	5.34 ± 1.68	
After 2 weeks	3.36 ± 1.13	3.22 ± 1.10	
Reduction	2.03 ± 1.52	2.12 ± 1.01	*P* = 0.776
*P* (baseline vs. 2 weeks)	<0.001	<0.001	
ALT			
Before treatment	724.79 ± 601.63	861.64 ± 691.44	
After 2 weeks	130.90 ± 278.18	119.51 ± 112.05	
Reduction	593.90 ± 540.26	742.13 ± 689.12	*P* = 0.294
*P* (baseline vs. 2 weeks)	<0.001	<0.001	
TBIL			
Before treatment	274.60 ± 138.61	259.64 ± 120.26	
After 2 weeks	239.89 ± 250.38	223.54 ± 124.94	
Reduction	34.71 ± 234.75	36.09 ± 105.37	*P* = 0.973
*P* (baseline vs. 2 weeks)	0.362	0.039	

**Table 3:** Comparison changes in serum creatinine and cystatin C between the ETV and TDF group.

	ETV (*n* = 39)	TDF (*n* = 39)	*P* (ETV vs. TDF)
sCr			
Before treatment	59.23 ± 11.24	57.86 ± 13.87	
After 2 weeks	61.06 ± 12.69	58.82 ± 11.56	
Changes from baseline to 2 weeks	−1.57 ± 5.95	−0.96 ± 10.32	0.748
*P* (baseline vs. 2 weeks)	0.080	0.565	
After 4 weeks	61.71 ± 12.14	60.92 ± 16.52	
Changes from baseline to 4 weeks	−2.68 ± 8.96	−2.17 ± 11.81	0.837
*P* (baseline vs. 4 weeks)	0.072	0.285	
Cystatin C			
Before treatment	1.06 ± 0.26	1.00 ± 0.21	
After 2 weeks	1.18 ± 0.32	1.11 ± 0.24	
Changes from baseline to 2 weeks	−0.12 ± 0.31	−0.11 ± 0.16	0.810
*P* (baseline vs. 2 weeks)	0.02	<0.001	
After 4 weeks	1.15 ± 0.16	1.28 ± 0.30	
Changes from baseline to 4 weeks	−0.08 ± 0.39	−0.25 ± 0.25	0.237
*P* (baseline vs. 4 weeks)	0.044	0.011	

**Table 4:** The clinical features of the AKI patients.

	A	B	C
Age	61	51	46
Sex	Male	Female	Male
sCr (baseline) (*μ*mol/L)	64	64	67
sCr (after treatment) (*μ*mol/L)	113	104	105
Antivirus therapy	ETV	TDF	TDF
Cirrhosis	Yes	Yes	Yes
Hypertension	Yes	Yes	No
Diabetes	Yes	No	No
Pneumonia	Yes	Yes	Yes
Outcome	Death	Survival	Survival

**Table 5:** Cox analysis of risk factors for mortality.

Variable	Univariate analysis (95% CI)	*P* value	Multivariate analysis (95% CI)	*P* value
Age	1.044 (1.010-1.079)	0.011	1.103 (1.038-1.172)	0.002
Male	1.276 (0.360-4.522)	0.706	1.200 (0.277-6.340)	0.830
HE	3.291 (1.123-9.644)	0.030	7.156 (0.740-69.170)	0.089
HBeAg-positive	8.356 (1.884-37.062)	0.005	10.611 (1.314-85.709)	0.027
Cirrhosis	1.253 (0.428-3.667)	0.681	47.232 (5.538-402.802)	<0.001
Antivirus therapy	0.885 (0.321-2.442)	0.814		
AKI	1.617 (0.213-12.302)	0.642	5.394 (0.535-54.420)	0.153
lgHBsAg (ng/mL)	0.874 (0.551-1.388)	0.570		
lgHBV-DNA (IU/mL)	0.920 (0.687-1.233)	0.577	0.925 (0.563-1.522)	0.760
ALT (U/L)	1.000 (1.000-1.001)	0.407		
sCr (*μ*mol/L)	0.985 (0.879-1.104)	0.795		
WBC (10^12^/L)	0.993 (0.857-1.151)	0.929		
PLT (10^9^/L)	0.997 (0.988-1.007)	0.598		
Meld-Na score	1.107 (1.020-1.201)	0.015	0.972 (0.837-1.128)	0.704
CTP score	1.743 (1.266-2.400)	0.001	1.990 (1.210-3.271)	0.007
SOFA score	2.146 (1.528-3.013)	<0.001	3.000 (1.621-5.553)	<0.001

## Data Availability

The data in this study are available from the corresponding author on reasonable request.
